# The Taxonomy of Opportunities to Learn (TxOTL): a tool for understanding the learning potential and substance of interactions in faculty (online) learning community meetings

**DOI:** 10.1186/s40594-021-00301-3

**Published:** 2021-07-17

**Authors:** Alexandra C. Lau, Makenna Martin, Adriana Corrales, Chandra Turpen, Fred Goldberg, Edward Price

**Affiliations:** 1 Center for Research on Instructional Change in Postsecondary Education, Western Michigan University, Kalamazoo, 49008-5288 MI USA; 2 Center for Research in Mathematics and Science Education, San Diego State University, San Diego, 92120 CA USA; 3 Department of Physics, University of Maryland, College Park, 20742 MD USA; 4 Department of Physics, California State University San Marcos, San Marcos, 92096 CA USA

**Keywords:** Faculty (online) learning communities, Opportunities for learning, Faculty professional development, Tool development, Research-based instructional strategies

## Abstract

**Background:**

While many research-based instructional strategies in STEM have been developed, faculty need support in implementing and sustaining use of these strategies. A number of STEM faculty professional development programs aim to provide such pedagogical support, and it is necessary to understand the activity and learning process for faculty in these settings. In this paper, a taxonomy for describing the learning opportunities in faculty (online) learning community meetings is presented. Faculty learning communities, meeting either in-person or (increasingly) online, are a common form of professional development. They aim to develop the pedagogical and reflective skills of participants through regular meetings centered on conversations about teaching and learning.

**Results:**

The tool presented in this paper, the Taxonomy of Opportunities to Learn (TxOTL), provides a structured approach to making sense of the dynamic interactions that occur during faculty learning community meetings. The origins and development of the TxOTL are described, followed by a detailed presentation of the constructs that make up the TxOTL: communicative approach used in a conversation, the concepts developed, and the meeting segment category. The TxOTL characterizes the learning opportunities presented by a faculty learning community conversation through describing the content of the conversation as well as how participants engage in the conversation. Examples of the tool in use are provided through an application to a faculty online learning community serving instructors of a physical science curriculum. A visual representation used to compactly display the results of applying the taxonomy to a meeting is detailed as well. These examples serve to illustrate the types of claims the TxOTL facilitates.

**Conclusions:**

The TxOTL allows one to examine learning opportunities available to a faculty learning community group, analyze concept development present in their conversations, track change over time in a given group, and identify patterns between meeting segment categories and communicative approaches. It is useful for researchers as well as facilitators of these STEM faculty professional development groups. The taxonomy is most applicable to faculty (online) learning communities, with limited use for workshops and K-12 professional development contexts.

**Supplementary Information:**

The online version contains supplementary material available at (10.1186/s40594-021-00301-3).

## Introduction

Over the last 30 years, the discipline-based education research (DBER) community has developed numerous research-based instructional strategies (RBISs) and materials and demonstrated their effectiveness at improving student conceptual understanding, engagement, and persistence in science, technology, engineering, and math (STEM) fields (Meltzer and Thornton 2012; Freeman et al. 2014; Hake 1998; Thiry et al. 2019). Faculty members are tasked with implementing these strategies, and there have been a number of national calls for STEM faculty to increase their adoption of RBISs (National Research Council (U.S.) Committee on the Status 2012; Olson et al. 2012). Research has shown that there is widespread knowledge of and motivation to try these research-based techniques and curricula among faculty, but that this has not translated into a commensurate level of sustained use (Henderson et al. 2012; Henderson et al. 2010). Faculty faces a number of barriers when implementing new teaching practices including limited time, physical space constraints of their classroom, and lack of training (Fairweather 2008; Brownell and Tanner 2012; Shadle et al. 2017; Dancy and Henderson 2010). It follows that faculty need more support in implementing RBISs and navigating the situational constraints they face (Henderson et al. 2015; Khatri et al. 2016; Henderson et al. 2010; Henderson et al. 2011). In order to provide this support, a deeper understanding of faculty professional development environments is essential.

There are a number of professional development programs for STEM faculty targeting their pedagogical development. For example, various STEM disciplines offer workshops for new faculty that, among other topics, introduce them to evidence-based teaching techniques (American Association of Physics Teachers 2021; American Chemical Society 2021; National Association of Geoscience Teachers 2019; Hilborn 2012). Faculty learning communities (FLCs) are offered at numerous institutions to support the teaching growth and course transformation for small groups of faculty over an extended (year-long) period of time (Cox 2004). These professional development programs serve as learning environments for faculty seeking to develop their pedagogical skills.

Given the role of professional development programs in supporting faculty members’ pedagogical growth, there is a need to not only study the effects of professional development programs on faculty (e.g., sustained implementation of RBISs, growth in skills such as reflection, formation of a supportive teaching community), but also to identify the structures and mechanisms in these learning environments that lead to these outcomes for faculty participants. In other words, a way to characterize what occurs during a professional development program is needed to better understand faculty learning in these settings. The nature and quantity of social interactions during any professional development program are complex and abundant, so being able to describe these interactions and specify their function and quality is informative for the design and facilitation of current and future programs (Manduca 2017). There are a number of studies that explore these topics in the context of K-12 teacher professional development (Turner et al. 2018; Horn 2010; Horn and Kane 2015; Lynch et al. 2019; Garet et al. 2001). Outcomes from STEM professional development programs in the context of higher education, such as changes in teaching practice and increased knowledge of pedagogical principles, have been reported (Chasteen et al. 2016; Henderson 2008; Manduca et al. 2017; Ebert-May et al. 2011; Borda et al. 2020; Derting et al. 2016), but there has been a lesser focus on the process that leads to these outcomes ((Tinnell et al. 2019; Olmstead and Turpen 2016; Turpen et al. 2018; Corrales et al. 2020) are exceptions). In this paper, we present a new tool that can be used to analyze the social interaction and processes occurring in a faculty learning community (FLC).

### Faculty online learning communities (FOLCs)

The context for the development of this tool is a faculty learning community that meets virtually. Called a faculty *online* learning community (FOLC), this is one form of faculty professional development that has the potential to support instructors in developing their pedagogical skills and sustaining implementation of research-based instructional strategies. The FOLC model translates the idea of an in-person faculty learning community (Cox and Richlin 2004; Tinnell et al. 2019; Thompson et al. 2016) to an online setting, connecting a group of approximately ten faculty members via periodic, hour-long video conference meetings and an asynchronous communication platform (e.g., Slack). The members of a FOLC are geographically distributed but are connected by a common teaching situation or interest. A FOLC group can be cohort-based (e.g., new faculty members; faculty members at small, teaching-focused institutions) or topic-based (e.g., members implementing the same curriculum or a certain teaching strategy). A FOLC is facilitated by faculty members who are experienced with the topic of the FOLC. More details on the origins and structure of the FOLC model as well as similar professional development programs can be found here (Bali and Caines 2018; Hayward and Laursen 2018; Pelletreau et al. 2018; Gehrke and Kezar 2019; Dancy et al. 2019).

During the online video conference meetings, FOLC members share successes and challenges related to the focus of their FOLC group and troubleshoot issues they are facing. The meetings are designed to be discussion-based, as opposed to a one-way transmission of information. These conversations are intended to be collaborative and supportive, encouraging members as they work through problems of practice (i.e., problems rooted in classroom experiences involving students, instructors, and/or content (Horn and Little 2010)). Discussion topics can range from pedagogical issues to professional concerns not directly related to teaching to larger social factors that are affecting faculty and students (e.g., the COVID-19 pandemic).

There is evidence that FOLCs are effective at supporting faculty in trying and persisting in using research-based teaching strategies and materials, and in reflecting on their teaching practice (Dancy et al. 2019; Corrales et al. 2020; Price et al. 2021). That said, not all FOLC groups unfold in identical ways, and their norms and conversational patterns can be quite distinct (Lau et al. 2019; Corrales et al. 2018; Turpen et al. 2018). Some groups focus on very practical implementation details whereas other groups engage in discussion of *why* certain problems are occurring and connect particular problems to more general phenomena. While both types of discussions can be appropriate and valuable, they offer different learning opportunities. In order to best support faculty members’ teaching development, we need to be able to describe the learning possible for faculty members in a given FOLC group as well as explain the variation in FOLC enactment across groups and within a group over time. Thus, a mechanism both for describing what occurs in a FOLC and for constructing claims about why it is happening will be useful in understanding the outcomes for faculty participants and guiding facilitation of these programs.

In this paper, we present a taxonomy we developed to describe the learning opportunities in FOLC meetings; we call this the Taxonomy of Opportunities to Learn (TxOTL). This tool provides a systematic approach to making sense of the dynamic and abundant social interaction that occurs during the hour-long FOLC meetings. We first describe the theoretical commitments underlying the TxOTL, specifically our choice to focus on capturing the opportunities to learn for the members of a FOLC group, collectively. We then detail the development process of the taxonomy, present the TxOTL itself, and introduce the elements of the TxOTL, with illustrative examples provided for each construct. Next, we discuss the utility of the taxonomy and demonstrate the types of claims the tool can help build, along with a discussion of the affordances and limitations of the tool. The TxOTL has both analytic and practical uses. We end by considering the contexts beyond a FOLC in which the TxOTL has use. While the TxOTL was developed in the context of a FOLC, we expect it to have broader applicability.

## Theoretical framework

### Opportunities to learn and a sociocultural perspective of learning

The taxonomy presented in this paper is built to describe the opportunities to learn (OTLs) in FOLC conversations. The focus on OTLs fits the need described in the “Introduction” section to attend to the process of learning and mechanisms contributing to learning (rather than only the outcomes) in these professional development spaces. Using OTLs as the framework of our tool allows us to make claims about how the environment and social structures of the FOLC contribute to learning of the collective group.

Our definition of OTLs is grounded in a sociocultural view of learning. Rather than viewing learning as a purely cognitive, individual process, a sociocultural perspective takes into account the effect of the environment (i.e., context, tools, culture), social interactions with others, and history (i.e., one’s prior knowledge) on the process of learning (Vygotsky 1978; Gee 2008; Engeström 2015; Lave and Wenger 1991; Brown et al. 1989). In this perspective, learning by an individual can be defined as changing participation in a community of practice (CoP), while learning by the community as a whole can be defined as a change in its practices (Vygotsky 1978; Lave and Wenger 1991; Greeno and Gresalfi 2008; Horn et al. 2017; Wenger 1998). As James Gee explains, “the central ideas [of a CoP] are that people learn new practices *through participation with others*, that they are networked with others and with various tools and technologies in ways that allow them to accomplish more than they could by themselves, and that *knowledge is stored as much in the network and the practices of the group as it is in any one person’s head*” (Gee 2008) [p.92, emphasis added]. In other words, learning is a social activity that is the product of interaction between an individual and their environment, and knowledge is distributed across these elements (Brown et al. 1989). A sociocultural perspective of learning, with its focus on social interaction and learning with others, undergirds the design of FOLCs. This theoretical grounding motivates the construction of FOLCs as CoPs, with a joint enterprise (i.e., domain they are gaining expertise in) of improving their teaching practice and a shared repertoire (i.e., ways of communicating, tools, artifacts) for accomplishing this goal (Lave and Wenger 1991; Wenger 1998; Wenger et al. 2002). The members of a CoP work together following a principle of mutuality, a negotiated means of engagement and interaction (Wenger 1998; 2000). With the perspective of learning as influenced and mediated by the environment and other people, changes in an individual’s interaction with these elements constitutes a change in participation, signaling that learning has occurred.

#### Group learning

With the Taxonomy of Opportunities to Learn (TxOTL), we consider the opportunities to learn available to the *FOLC group* (Horn and Kane 2015; Horn et al. 2017). Our unit of analysis is a FOLC conversation, and this positions us to make claims about the OTLs for the *collective* participants in the conversation, rather than the *individual* members of the conversation. (Note, an hour-long FOLC meeting consists of a number of distinct conversations.) One can view a conversation as representing a collective zone of proximal development (ZPD) for the group engaging in the conversation (Engeström 2015; Horn and Kane 2015). As originally defined by Vygotsky, an *individual’s* ZPD represents the developmental tasks or functions they can achieve with the assistance of others and are in the process of being able to accomplish on their own (Vygotsky 1978). The concept of a ZPD helps describe the trajectory and future of one’s development because it “defines those functions that have not yet matured but are in the process of maturation” (Vygotsky 1978) [p.86]. When focusing on the learning of a group rather than an individual, it becomes appropriate to consider a collective ZPD: the concepts, solutions, and ideas about what is possible (to learn and do) that are available to the collective group, but not necessarily the individuals who make up the group (Horn and Kane 2015; Engeström 2015). The conversations that a group engages in contain the conceptual resources available to the group, helping to define their collective ZPD (Horn and Kane 2015; Engeström 2015). It is through conversation that OTLs arise and are shaped for a FOLC group.

As previously discussed, individual learning can be identified when a community member changes their participation in the community; similarly, the learning of a group occurs when there is a “change in its practices” (Greeno and Gresalfi 2008) [p.174]. The practices of a group include the conceptual resources it draws on, the ways it approaches solving problems, and its discourse. Consider an example by Greeno and Gresalfi of a class learning: “A classroom’s practices change as information and concepts are added to its common ground, supporting changes in the content of its discourse...the practices of the classroom, specifically in terms of the ways participants can make sense of new information, change. Opportunities to learn for a classroom include resources and practices that can support the extension and transformation of those practices” (Greeno and Gresalfi 2008) [p.175]. In other words, when ideas are introduced in the classroom, they provide additional ways for the class to understand concepts and phenomena, and when the class enacts these ways of understanding that constitutes a change in its practice. We can draw a parallel from this classroom example to FOLC conversations; as faculty discuss their teaching with a FOLC group, different ways of thinking about a pedagogical issue are introduced and a range of concepts are brought into the conversation. When these resources are new to the group as a whole, its collective ZPD expands as its sense of what is possible (in terms of teaching) grows. If these concepts and ways of thinking are later taken up and employed by the group, that constitutes a change in practice (and thus learning) for the group.

#### Defining opportunity

When we talk about OTLs, we are using “opportunity” in a literal sense: an OTL describes the *potential* for learning to occur. OTLs are “affordances for changing participation and practice” (Greeno and Gresalfi 2008) [p.172], where affordances describe the resources and practices of a community, and the ability of members to use the resources and interact with the practices (Greeno and Gresalfi 2008; Norman 1988; Gibson 1979). The TxOTL describes the affordances of FOLC conversations for changing how the group acts and thinks about itself, the practice of teaching, and different pedagogical challenges they face.

#### Operationalizing opportunity to learn

We operationalize OTL by considering what causes a change in participation. Following Horn and colleagues’ definition, we describe an OTL provided by a FOLC conversation by considering the conceptual resources employed in the conversation, how the conversation prepares participants for their future work, and how ideas were communicated in the conversation (Horn and Kane 2015; Horn et al. 2017; Horn et al. 2015). Conceptual resources include ways of representing one’s practice (e.g., replays and rehearsals), ways of interpreting those representations, different problem framings, and epistemic stances (Horn and Kane 2015; Horn 2010; Goffman 1974; Hall and Horn 2012). These resources are what a group uses to understand a problem and to imagine possible solutions.

For the purpose of the TxOTL, we describe both pedagogical and non-pedagogical OTLs in FOLC conversations. We define pedagogical as a conversation attending to students’ learning and the effects of teaching practices on their learning. In a FOLC, OTLs extend beyond the pedagogical to include other learning opportunities (e.g., the opportunity to get to know fellow group members) which affect the ways the community functions.

## Methods

### Development context

The TxOTL was primarily developed in the context of the Next Generation Physical Science and Everyday Thinking (NextGenPET) FOLC (Price et al. 2021). Given the known challenges to implementing research-based instructional strategies (Henderson et al. 2011; Henderson et al. 2010; Shadle et al. 2017; Khatri et al. 2016), the NextGenPET FOLC was established to support instructors who are implementing the NextGenPET curriculum (Goldberg et al. 2018). NextGenPET is a physical science curriculum for future elementary teachers that, at times, is also used in general education university science courses. The curriculum employs a guided-inquiry pedagogy, allowing students to collaboratively construct the main ideas in each unit through experimentation and modeling. NextGenPET is aligned with the Next Generation Science Standards (NGSS Lead States 2013). Instructors can choose their implementation format based on their classroom constraints (either lecture or studio-style course^1^). Instructors also choose the topics to cover from five modules: developing models for magnetism and static electricity; interactions and energy; interactions and forces; waves, sound, and light; and matter and interactions.

There are five NextGenPET FOLC groups, each consisting of approximately ten members who are teaching the curriculum, including two facilitators who are experienced NextGenPET practitioners. The immediate goal of the NextGenPET FOLC is to support instructors’ implementations of the curriculum; a longer-term goal is to help FOLC members apply the pedagogical knowledge, skills, and research-based techniques they learn from teaching NextGenPET to the other courses they teach. In their FOLC video conference meetings, members are encouraged to reflect on their practice. Their shared curricular context affords unique learning opportunities as FOLC discussions can cover curriculum-specific topics such as difficulties students are encountering with a particular NextGenPET activity. In addition to these NextGenPET-specific topics, the FOLC groups also discuss more general pedagogical issues, like how to handle student groups who work at different paces. From monitoring a number of FOLC meetings across various groups, we know the content and norms of their conversations are not identical. Each group’s meetings are focused on implementation of the NextGenPET curriculum and challenges members are facing, but the types of issues they focus on and the nature of their conversations vary. We created the taxonomy as a mechanism to describe the OTLs in a FOLC group meeting; the information gained by applying the taxonomy to different FOLC meetings can assist in a comparison of the FOLC groups and can help explain the differences in a group’s meetings over time.

### Taxonomy origins

The TxOTL is adapted from an existing framework for classifying opportunities to learn about teaching (Horn et al. 2017). Horn, Garner, Kane, and Brasel constructed a taxonomy to describe how different forms of interaction afford different types of learning in the context of in-person, middle school mathematics teacher workgroups. More specifically, their taxonomy describes how collegial conversations range in the nature and depth of support they provide for teachers’ learning (Horn et al. 2017). The framework defines six categories of workgroup meetings based on the pedagogical concepts developed, the degree of mobilization for future teaching work, and the nature of the discourse in the meeting. We used this framework as a starting point for analyzing NextGenPET FOLC meetings.

Horn et al.’s taxonomy characterizes OTLs by considering both the *content* of a conversation as well as *how* participants engage in the conversation (Horn et al. 2017). They see the richest learning opportunities as occurring when a conversation involves the teachers developing a pedagogical concept while mobilizing them for their future teaching work. The TxOTL preserves the overall organization of Horn et al.’s taxonomy, but the outcome of applying that organization to FOLC meetings has resulted in a taxonomy that differs substantially from theirs.

### Tool development process

We began by applying Horn et al.’s taxonomy (Horn et al. 2017) to (the transcript of) one NextGenPET FOLC meeting in order to see how well the taxonomy fits the FOLC context. This initial test alerted us to a number of changes that would be required to adapt the taxonomy to our context. For example, the meeting categories which comprehensively describe the types of mathematics teacher workgroup meetings in Horn et al.’s data corpus did not do the same for FOLC meetings. While some of their categories applied to the FOLC context, many did not. For example, Horn et al.’s “Collective Interpretation” categories describing when a teacher workgroup develops a pedagogical concept did apply to the FOLC context where conversation about problems of practice can lead to concept development within the group. In contrast, one of the categories in Horn et al.’s taxonomy is “Pacing” which describes conversations centered on coordinating the timing of future lessons; this did not apply to the FOLC context because unlike the middle school mathematics teachers Horn et al. studied, FOLC members are located at different institutions and have no need to coordinate their instruction. It was clear we would need to define additional meeting categories to capture the full scope of FOLC meetings. We also wanted to describe *how* participants engage in a conversation in more detail than Horn et al.’s taxonomy provides. Influenced by the categories used in a tool to study professional development workshops (the R-PDOT (Olmstead and Turpen 2016)), we turned to the work of Scott, Mortimer, and Aguiar (Scott et al. 2006) for constructs that would add the desired level of detail.

We went on to apply the taxonomy to three additional NextGenPET FOLC meetings, iteratively refining the tool with each application. One member of the research team (author A.C.L.) would divide a meeting based on shifts in conversational purpose (Schegloff 2007). Then, that member and two to three additional members of the research team independently coded each meeting segment along the dimensions of the taxonomy (describing both the content of the conversation and how the conversation unfolded). Each time the researchers were working from a codebook which represented the current version of the TxOTL). The team of coders would then compare their coding for each segment and iterate until full agreement was reached. This comparison was guided by comparing the segment-in-question to touchstone examples of a code as well as contrasting cases (Maxwell 2013); this ensured consistency with our prior coding and added robustness to our definitions. Through these coding comparisons, we specified the code definitions and resolved the gaps and redundancies in the framework. As the TxOTL evolved, we re-coded the meeting segments as necessary to reflect any definition changes we had made.

As will be described in “Results: the Taxonomy of Opportunities to Learn (TxOTL)” section, the three main dimensions of the TxOTL are communicative approach, concept development, and meeting segment category. When comparing our coding, disagreements arising from two code categories (communicative approach and meeting segment category) were often due to the differing understandings of the code definitions. These disagreements were resolved by clarifying the definition of a code (and adding that additional guidance to the codebook). In contrast, disagreements arising from a third coding category (concept development) took more negotiation and discussion to resolve. This is perhaps unsurprising as this category involves identifying concepts, and historically, the whole notion of what constitutes and counts as a concept has been widely debated (diSessa 1993; Smith III et al. 1994). Following (sometimes lengthy) discussion, we were able to reach consensus, and we added guidance to our codebook to help in coding similar segments in the future. The process of discussing coding disagreements and coming to consensus was valuable because we gained a deeper understanding of the taxonomy elements and how they apply. These coding discussions also served as a check on over-interpreting the dialog as we had to be able to justify our interpretation (and the coding that followed) to the other coders.

During the TxOTL’s development, a new researcher joined the team and was able to gain a functional understanding of the tool in a short amount of time after doing some background reading of the literature we draw on (i.e., Horn et al. (2017); Scott et al. (2006)) and participating in a few training sessions. In the training sessions, she practiced applying the taxonomy to a FOLC meeting and comparing her coding to that of more experienced team members. This training experience, as well as all the other coding comparison discussions among the research team, has resulted in a codebook which has “touchstone examples” for some of the codes (i.e., specific segments from a FOLC meeting that helped us define the scope of a code); common phrases that appear in segments coded certain ways; as well as documentation of our process for handling tricky cases. The information contained in the codebook is incorporated throughout “Results: the Taxonomy of Opportunities to Learn (TxOTL)” section, where the taxonomy is presented.

We purposefully chose the four NextGenPET FOLC meetings used in developing the TxOTL to come from two groups with different norms because this captured a wider range of the interactions that can occur in these types of professional development settings. This work resulted in the first complete version of the TxOTL (see Table 1 for the version history of the TxOTL). Through this cycle of coding and comparison, we developed our definitions for how to describe the content of a conversation (encoded in concept development codes and meeting segment category codes) and for how to describe the discursive nature of a conversation (encoded in communicative approach codes).

**Table 1:** Summary of the versions of the TxOTL and the FOLC meetings on which each version was tested

Version	FOLC meetings applied
V 1.0 (Horn et al. 2017)	1 NextGenPET FOLC meeting (meeting A)
V 1.1	3 NextGenPET FOLC meetings (meetings B–D)
V 1.2 *(first complete version)*	2 NFW-FOLC meetings
V 1.3	2 NextGenPET FOLC meetings (meetings A and B)
V 2.0 *(second complete version)*	0 FOLC meetings; feedback gathered from member-checking interviews
V 2.1 *(final version)*	N/A

### Tool refinement

Our next goal was to test the applicability of the first complete version of the TxOTL to other FOLC groups, as we wanted the tool to be useful beyond the specific NextGenPET FOLCs. The New Faculty Workshop FOLC (NFW-FOLC) provided the context for this test. The NFW-FOLC connects new physics and astronomy faculty for the year following their attendance at the in-person Workshop for New Physics and Astronomy Faculty (American Association of Physics Teachers 2021). The Workshop introduces faculty to research-based instructional strategies, and the FOLC supports the faculty members as they implement a range of these techniques in their classrooms. The structure of the NFW-FOLCs is somewhat similar to that of the NextGenPET FOLC (e.g., frequent video conference meetings to discuss teaching and an asynchronous platform to connect between meetings). However, members of the NFW-FOLC are exclusively new physics and astronomy faculty (rather than the mix of career stages and STEM departments the NextGenPET FOLC members come from), and they are not using the same curriculum. NFW-FOLC members are implementing a range of named RBISs in their classrooms (e.g., peer instruction (Mazur 1997; Crouch et al. 2007), just in time teaching (Novak et al. 1999; Patterson 2004), lecture-tutorials for introductory astronomy (Prather et al. 2004; Prather et al. 2013)), as well as more generalized active learning strategies (e.g., small group work). While all of these techniques promote the active engagement of students, the NextGenPET curriculum has unique pedagogical commitments with a focus on guided-inquiry, experimentation, and model building. All this is to say that while the two FOLCs share a number of similarities, we might expect their different foci to result in conversations that present some distinct types of OTLs. Therefore, the NFW-FOLC was a useful context in which to test the applicability of the preliminary version of the TxOTL.

We applied the TxOTL to two NFW-FOLC meetings following the same process as described above: individual coding followed by comparison and discussion until consensus was reached. The taxonomy largely applied to the context of the NFW-FOLC, but we found that we needed to refine meeting segment category and communicative approach definitions to account for the slightly different conversational routines and content that occur in the NFW-FOLC.

At this point, the taxonomy had changed significantly since the beginning of the development process. Therefore, we returned to the first two NextGenPET FOLC meetings we had coded to re-code based on the current TxOTL version. With this last round of coding, we finalized the second version of the TxOTL.

In developing the TxOTL, we purposefully chose to analyze meetings that came from two NextGenPET FOLC groups with different conversational norms, as well as meetings from the NFW-FOLC (given its different focus from that of the NextGenPET FOLCs). The variety in our selected meetings helped us ensure that we were capturing the range of interactions that can occur in a FOLC meeting, thus allowing the taxonomy to have the breadth and flexibility necessary for application across different FOLC meetings and groups. We were able to choose meetings representing a range of interactions and discussions based on our regular monitoring of the FOLC meetings (many more than the six analyzed in developing the TxOTL). The meetings chosen for analysis were illustrative of the different types of FOLC meetings we had observed. Our familiarity with the NextGenPET FOLC and the NFW-FOLC allowed us to determine that the six meetings used to develop the TxOTL sufficiently captured the variability in FOLC meetings. Of course, in *applying* the tool one may need to use more than six meetings depending on the claims they are trying to make (this is discussed further in “Interpreting a timeline representation” section), but for *building* the tool, six meetings captured the necessary variety.

### Member-checking interviews

As a final step in the development process, we presented the second full version of the TxOTL to four NextGenPET FOLC facilitators and interviewed them about their perspectives on the usefulness of the tool. We chose our facilitator sample purposefully to (1) overlap with the interests of other research strands within the NextGenPET project, (2) hear from facilitators of different NextGenPET FOLC groups, and (3) talk to those with distinct approaches to facilitating. To start the member-checking process (Maxwell 2013), we sent the facilitators a five-page document to review which provided an overview of the taxonomy: our motivation for making it, the intended purpose of the tool, the definitions of all the taxonomy elements, and a visual representation (detailed in “Timeline representation” section) of the results of applying the taxonomy to a FOLC meeting. At the beginning of the interviews, facilitators were asked if they had clarifying questions about the taxonomy. This ensured that we established a shared understanding of the definitions, structure, and overall purpose of the taxonomy. Next, we asked about the information provided by the visual representation of the results of applying the taxonomy to a FOLC meeting, and we asked them to postulate what was occurring in the meeting based on their interpretation of the representation. We asked how the representation could inform their practice if they were the facilitator of the meeting shown, and if the representation was lacking information about aspects of a meeting that they find important. We then presented a representation of a second meeting coded with the taxonomy and asked about the differences they saw and the inferences they could make in comparing the meetings.

The feedback from the four facilitators alerted us to both affordances and constraints of the taxonomy. Briefly, the facilitators felt that overall the TxOTL applied to a FOLC meeting provided interesting information that could inform their practice. Their interpretations based on the taxonomy applied to two meetings were mostly inline with those of the research team. The TxOTL was designed to make claims about a group’s OTLs, and to capture finer-grained differences within pedagogical OTLs, but the FOLC facilitators were also interested in the names and number of people talking during a meeting segment; the type of social talk engaged in; and the specific number of ideas raised during a conversation. They also were curious about certain conversational patterns that transcend meeting segment categories. These details are beyond the scope of the TxOTL.

The facilitators also offered a number of suggestions on how the visual representation of the results of applying the taxonomy to a meeting could be improved. One of the facilitators suggested a different label for one of the communicative approach categories that they thought would better illustrate the meaning of that code; we modified the taxonomy to include this new label. After incorporating the feedback from facilitators into the TxOTL, we had a final version of the tool. Table 1 summarizes the version history and development of the TxOTL.

## Results: the Taxonomy of Opportunities to Learn (TxOTL)

The TxOTL is organized around three major constructs: communicative approach, concept development, and meeting segment category. Communicative approach describes *how* people engage in a conversation. Concept development coding identifies the pedagogical concepts people introduce into a conversation, while meeting segment category describes the broad topic and function of a conversation. Together, concept development coding and meeting segment category coding describe *what* people are talking about.

**Fig. 1:**
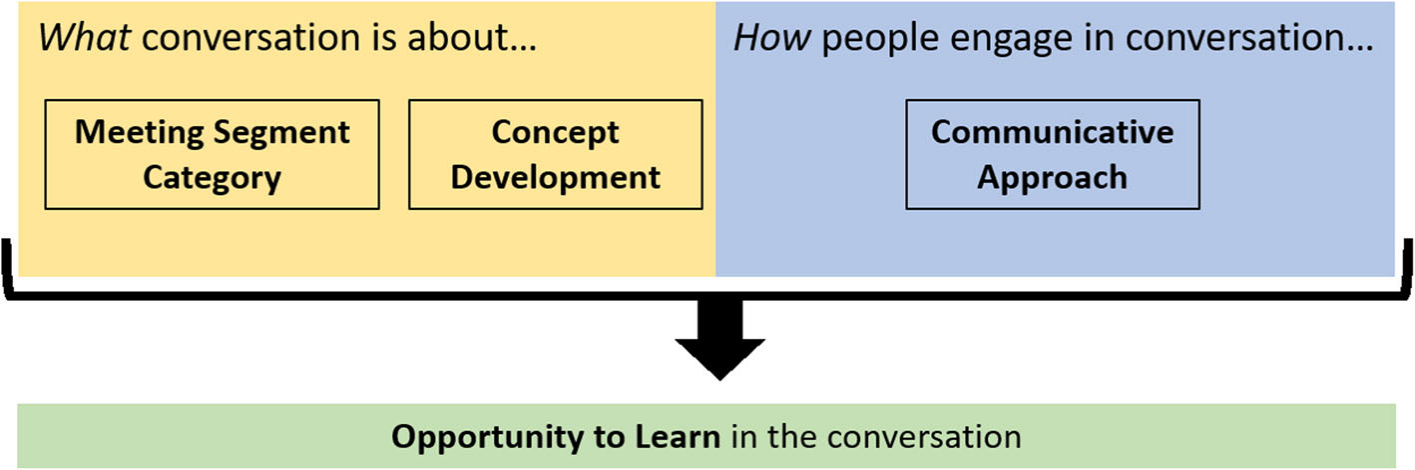
Depiction of the main structural elements of the taxonomy for describing opportunities to learn (the TxOTL). Meeting segment category and concept development help describe the content of a conversation, while the communicative approach describes how people engage in the conversation. Together, these three elements describe the opportunity to learn provided by a conversation

**Fig. 2:**
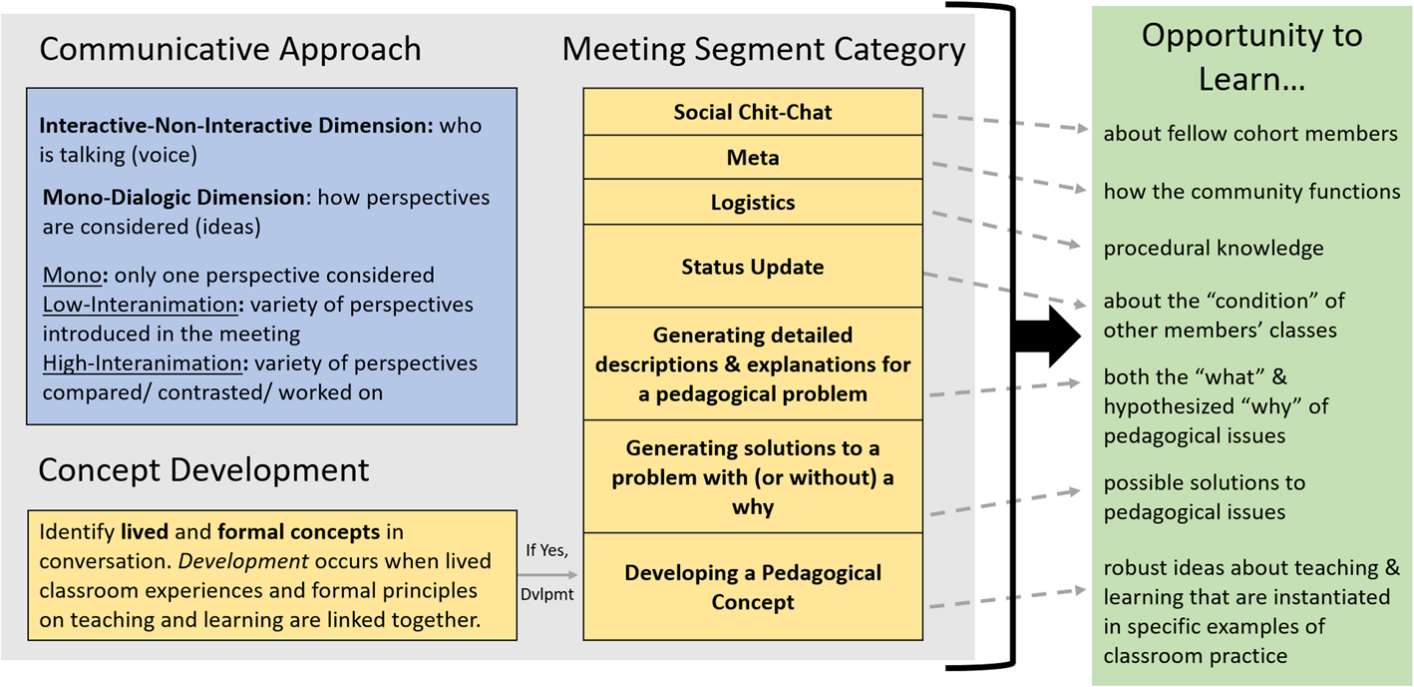
Detailed overview of the taxonomy for describing opportunities to learn (TxOTL) in FOLC meetings. A meeting segment is coded for communicative approach, concept development, and meeting segment category. Communicative approach is described by subcodes detailing who is talking and how perspectives (ideas) are considered. In the taxonomy, coding for concept development is restricted to pedagogical concepts; if it is determined that pedagogical concept development occurred in a segment, that segment gets labeled with the meeting segment category of Developing a Pedagogical Concept. If there is no pedagogical concept developed, the segment gets labeled as the appropriate one of the other 7 meeting segment categories. The dashed arrows indicate that the OTL in a meeting segment can be *broadly* characterized by the segment’s meeting category. The solid bracket signifies that in order to describe an OTL most accurately and in *detail*, all three taxonomy elements must be considered

A FOLC meeting can be divided into conversational segments based on the purpose of a conversation; a shift in purpose begins a new conversational segment (Schegloff 2007). Segments can last less than a minute to over 20 min. The taxonomy is meant to be applied to each segment (i.e., each segment is coded for communicative approach, concept development, and meeting segment category). Together, these three elements describe the OTL provided by a meeting segment. Once the taxonomy is applied to each segment of a whole meeting, one is provided with a detailed description of the OTLs constructed in the hour-long meeting. Figure 1 provides an overview of the structural elements of the TxOTL, and Fig. 2 provides a more detailed picture of the framework. Below, we describe each of the major elements of the taxonomy and provide examples for each code.

### Communicative approach

An important aspect of the social interaction in FOLC meetings is how members are communicating with each other. The communicative approaches used are consequential for the learning opportunities provided by a conversation. In order to describe how people are engaging in FOLC meetings, we adapted Mortimer and Scott’s classification scheme for describing classroom talk (Mortimer and Scott 2003; Scott et al. 2006). This scheme defines communicative approach along two dimensions: classifying who is talking (the “interactive-non-interactive” dimension) and classifying how ideas are discussed (the “mono-dialogic” dimension).

#### Interactive-Non-Interactive dimension

*Interactive* talk means there is substantive engagement in the content of the conversation by more than one person. This can range from a person asking clarifying questions about an idea someone else shared to (multiple) people adding ideas, solutions, or reflections on the topic being discussed. In contrast, *non-interactive* talk describes conversations in which only one person is substantively contributing. Other people can talk during a non-interactive conversation, but their contributions stay at the surface level, limited to simple phrases of agreement or acknowledgement (e.g., “I agree,” “umm hmm”) or a facilitator directing the conversation (e.g., “Sue, go ahead”).

#### Mono-Dialogic dimension

This dimension describes *how* ideas are discussed and taken up; it has nothing to do with the number of voices in the conversation. *Mono* conversations consider one perspective (idea) on an issue/topic or involve the sharing of facts. A segment where two or more ideas are mentioned, but they are not related to the same issue (hence, they cannot be compared, contrasted, or developed) is also considered mono. *Dialogic* conversations involve the consideration of multiple perspectives on a single issue/topic.

Dialogic discourse can be further described based on how the multiple perspectives are introduced into the conversation; this is known as the *interanimation* of ideas (Bakhtin 1981; Scott et al. 2006). Dialogic discourse involves *low-level interanimation* of ideas when, “different ideas are made available on the social plane [of the FOLC meeting]” (Scott et al. 2006) [p.611]. Dialogic discourse involves *high-level interanimation* of ideas when, “different ideas are explored and worked on by comparing, contrasting, developing” (Scott et al. 2006, p.611). With low-level interanimation, ideas are simply introduced to the conversation, while with high-level interanimation, those ideas are directly connected through comparison and/or development.

The distinction between mono discourse, dialogic discourse with low-level interanimation of ideas, and dialogic discourse with high-level interanimation of ideas is most easily understood through an example. In Table 2, we use the example of a discussion on how people implement attendance policies in their classes. The examples also demonstrate how the mono-dialogic dimension of communicative approach is independent of the interactive-non-interactive dimension; it is possible for one person to produce dialogic talk.

**Table 2:** Examples of mono discourse, dialogic discourse with low-level interanimation of ideas, and dialogic discourse with high-level interanimation of ideas

How ideas are discussed	Example
**Mono**	A. Somebody shares how they enforce attendance in their class, “I do X.” This is not followed up on; conversation moves on to a new topic.
	B. *Sharing facts:* someone says, “My institution has a policy which prohibits us from including attendance in our grades.”
	C. Somebody shares how they enforce attendance in their class, “I do X.” Other people ask clarifying questions about the policy, e.g., “Can you say more about X?”
	D. *Two incomparable ideas:* somebody shares how they enforce attendance in their class, “I do X.” They then add, “But you know, attendance doesn’t seem to affect students’ performance on exams.” This is not followed-up on by anyone else.
**Dialogic discourse with low-level interanimation of ideas**	E. Multiple people share how they enforce attendance in their class. This is done in a round-robin format: “I do X,” “I do Y.” People do not directly engage in what others have shared.
	F. One person shares “I used to do X to enforce attendance. This semester I am using Y.” They do not directly compare these practices.
**Dialogic Discourse with high-level Interanimation of ideas**	G. Multiple people share how they enforce attendance in their classes and these ideas are directly compared, contrasted, and/or engaged with. Person A shares, “I do X.” Person B responds, “I’ve tried X before and it didn’t work for my class because of ___. Instead, I find that practice Y is a more effective strategy.”
	H. One person shares, “I used to do X to enforce attendance and I thought it was perfect for my small class size. However, this semester I also have a small class and X has not worked. Instead, I now think Y is a better strategy to handle attendance in small classes because ___.”

Together, the two dimensions that describe how people are talking combine to form six options for characterizing the communicative approach of a meeting segment (see Table 3). These communicative approaches (specifically the mono-dialogic dimension) help describe the OTL provided by each meeting segment. As the examples in Table 2 show, the mono-dialogic dimension of the communicative approach tells whether one or more than one perspective is considered regarding the topic of conversation and how these perspectives are attended to. Every conversation during a FOLC meeting provides an OTL, regardless of the communicative approach used, but the mono-dialogic dimension of the communicative approach helps us describe the depth and scope of that OTL. Following the examples in Table 2, the mono-dialogic dimension of the communicative approach can tell us how many ideas about enforcing attendance were shared during a conversation and how those ideas were discussed (i.e., were the ideas taken up, compared).

**Table 3:** The six communicative approaches. The examples noted in parentheses refer to the examples listed in Table 2

	Interactive	Non-interactive
**Mono**	Interactive/mono	Non-interactive/mono
	*(example C)*	*(examples A, B, and D)*
**Dialogic-low-level interanimation**	Interactive/dialogic-low-interanimation	Non-interactive/dialogic-low-interanimation
	*(example E)*	*(example F)*
**Dialogic-high-level interanimation**	Interactive/dialogic-high-interanimation	Non-interactive/dialogic-high-interanimation
	*(example G)*	*(example H)*

Communicative approach also helps us from a practical, meeting-facilitation perspective. In addition to the information that can be gained from the mono-dialogic dimension, the interactive-non-interactive dimension helps answer the question, *Are multiple people contributing to a conversation, or only one person?* Ideally, the majority of segments within a FOLC meeting will be interactive, including the voices of multiple participants. (Recall, from a sociocultural perspective, learning is mediated by interactions with others.) Thus, communicative approach is one metric to evaluate if we are achieving this goal.

### Concept development

To begin to describe *what* a conversation is about, we consider what pedagogical concepts are present in the talk. We look for formal concepts and lived concepts; both are conceptual resources utilized in conversation. *Formal (pedagogical) concepts* are theories, abstractions, principles, or generalizations about teaching and learning (Horn et al. 2017). *Lived (pedagogical) concepts* are experiences in the world, for example, replays of things that have occurred in one’s classroom or rehearsals of teaching techniques one will try (Horn et al. 2017; Horn 2010). Table 4 includes examples of formal and lived pedagogical concepts (hereafter referred to simply as formal and lived concepts).

**Table 4:** Examples of formal and lived pedagogical concepts

Type of concept	Example
Formal pedagogical concepts: *theories, abstractions, principles, or generalizations about teaching and learning*	Ex 1: “Students are more comfortable asking a TA or LA for help because they are less intimidating than the professor.”
	Ex 2: “Students need to have agency over their learning.”
Lived concepts: *experiences in the world, for example, replays and rehearsals of classroom experiences*	Ex 1: “Students had a really hard time with activity 5 yesterday.”
	Ex 2: “I am not going to give unit tests this term. I will only give module exams.”

We examine the lived and formal concepts in a conversation in order to determine if the conversation involved the *development* of a pedagogical concept. We draw our definition of concept development from Horn et al. Horn et al. (2017), who in turn are applying a Vygotskian perspective (Vygotsky 1986). According to this perspective, as summarized by Horn et al., concept development occurs when conversation “bring[s] the general and the particular together—by surfacing the formal dimensions of lived concepts or illustrating lived examples of formal concepts” (Horn et al. 2017) [p.43]. Concept development means formal and lived concepts are linked in the course of a conversation. When we identify concept development in a conversation, the concept may not be new to individual participants and is generally not new to the larger education community; however, the concept emerges for the collective FOLC group through their teaching-focused conversation. In other words, the linking of formal and lived concepts is novel to the group as a whole and constitutes an expansion of their collective zone of proximal development. The linking of lived and formal concepts may be done by multiple participants or by an individual. We take the stance that even if the linking is done by an individual, because the development is verbalized in the meeting, it becomes available to the whole group. Additionally, concept development does not require everyone to come to agreement about the ideas shared; the important feature is that the ideas and connections are made available to the group members through the conversation.

Attending to pedagogical concept development is important for at least two reasons. Recall from the theoretical framing section (specifically “Operationalizing opportunity to learn” section) that OTLs can be operationalized by considering the conceptual resources and mobilization for future teaching work present in a given conversation (Horn and Kane 2015; Horn et al. 2017; Horn et al. 2015). A *variety* of conceptual resources are employed during pedagogical concept development (e.g., representations of practice, problem framings, and epistemic stances), providing a unique form of OTLs. Second, concept development mobilizes community members for a *range* of future teaching situations, not only the issue that sparked the conversation in which concept development occurred. Concept development provides the group with tools and pedagogical concepts that can be applied in a variety of contexts (in the NextGenPET course and others). Additionally, the act of linking lived and formal concepts often involves a substantive level of reflection from the group, and developing the reflective skills of NextGenPET FOLC members is one of the community’s goals (similar to that of other professional development efforts, e.g., Cox and Richlin (2004); Kember and McKay (1996)).

In many FOLC conversations, lived and formal concepts are present, but these concepts are not always joined in a way that would constitute concept development. In order to decide if a concept was developed, we employ the “engaged newcomer” heuristic (Horn et al. 2017). This heuristic causes one to consider a newcomer to the FOLC meeting. The newcomer is similar to the other FOLC members (e.g., they also teach the NextGenPET curriculum), but they are a novice to the FOLC meetings and to the topic of the FOLC (e.g., they are teaching NextGenPET for the first time). In reviewing a conversation, we ask ourselves, “would an engaged newcomer to the conversation have made a connection between these lived concepts and these formal concepts?” This heuristic prevents us from inferring too much from the conversation, based on our background knowledge of the topic being discussed or the speaker(s) doing the linking. We only say there was concept development if we think it is reasonable that an engaged newcomer to the conversation would have picked up on the developed concept.

It is perhaps easiest to understand what concept development means by seeing an example of what it looks like in practice.

In Table 5, we provide the transcript of an excerpt from one of the NextGenPET FOLC group’s meetings. Directly preceding this meeting excerpt, the group members are taking turns sharing updates from their NextGenPET classes. Wallace tells the group that his class is almost finished with the magnetism unit of the curriculum. In this unit, students are guided through an iterative process of prediction, experimentation, and revision as they construct a model for how magnetism works. Wallace describes how his students have trouble making predictions based on their working model, and the group discusses how to help students with the model building process. The conversation then shifts to a second, related issue Wallace has been facing. This new conversation, which lasts 4 min, is shown in Table 5.

**Table 5:** Transcript of a 4-min NextGenPET FOLC conversation about students searching the internet for answers. All names are pseudonyms. Formal concepts (FC) are in bold, and lived concepts (LC) are in italics. Turns of talk represent a continuous flow of conversation

Turn	Speaker	Transcript
1	Wallace	The other issue I have, which is... I think this mainly arises because we meet only twice a week for such a short time, so the module gets drawn out over a few weeks, is *some students go away and google how magnetism works, and so suddenly they’ll be talking about domains. Okay, so this is kind of skewing the process*.
2	Courtney	Yeah, *but I’ve found that in my experience anyway, if they go away and they come back with domains that they usually don’t have any idea how they actually work*.
3	Wallace	No, yeah, that’s true.
4	Courtney	*They try to use domains to explain whatever, and they can’t, and so the rest of the class is like, “Well, never mind that. We’ll just forget... ”*
5	Wallace	I think that is true. They google the answer, but they’re not really quite understanding what’s going on still. I’m not too worried about that. It was just funny when they suddenly start pulling out these words.
6	Carter	**Kind of seems like it’s evidence about the students’ epistemology, like I feel like they don’t have very sophisticated views about what it means to understand something**.
7	Courtney	Oh, they don’t.
8	Carter	**Because science context, what it means to understand something, and so for them understanding means like knowing the term or being familiar with the term when we’re trying to give them an experience that’s so much different view of what it means to understand something, and there’s a tension there**.
9	Courtney	*Yeah, they want to memorize. “I must memorize.”*
10	Carter	*I had a student after the quiz, he was in my office complaining, different student than the other one that I mentioned earlier, he’s going about how he understands everything in this class, because after all this class is like baby physics, and he learned it all in high school, but he just can’t explain it the way that I want him to. He went on and on and on and on. I tried to provide some, “Have you thought about maybe writing an outline of the key bullet points that you wanna hit in your explanation, and only then start... ”*
11	Carter	*I just tried everything I could to get him to reflect on maybe “I don’t fully understand it.***My struggles are evidence that I don’t fully understand it**.” *Every time I tried to hand it back to him, he just kept handing it back to me. Like, “No, this is so easy, and I just... yeah, I can’t explain it the way you want.” Oh God, just leave.*
12	Courtney	Right.
13	Yin	What is domain? I’m sorry I don’t think I fully understand. What kind of question that they google?
14	Wallace	Oh, *this is they’re googling... they’re basically trying to google how ferromagnetism and iron gets magnetized, so they come across the idea of magnetic domain, certain small regions that are polarized in the magnet*.
15	Wallace	**They generally don’t really understand what that means. They just start using these words because it’s something they’ve seen.**
16	Carter	Yin, have you taught the magnetism unit?
17	Yin	No, but I am very much looking forward to it.
18	Carter	Yeah, it’s so awesome. I would encourage you... you gotta find a way
19	Wallace	Despite these problems, and *I think it is really good, and I think the students are getting a lot out of it*.

Wallace shares his experience (LC, turn 1 in Table 5) that students will search the internet for how magnetism works, and he is concerned that this is “skewing the process” built into the curriculum. The magnetism unit takes several weeks for the class to work through and students are supposed to be constructing their model of magnetism based on evidence collected in class, rather than searching for the answer on the internet. Courtney responds to his concern with her own LC that students do not actually understand what they look up on the internet (turn 2). They may read about the domain model of magnetism, but they cannot actually explain it, so it is ignored by the class (turn 4). Wallace agrees with Courtney’s comment that students do not understand what they find online, and this seems to assuage his concern (turn 5).

Carter offers an explanation for this situation with the formal concept of epistemology (turn 6). He suggests that for students, “understanding means like knowing the term,” whereas in science, understanding entails something deeper than being familiar with terminology (turn 8). Courtney agrees with this interpretation, saying her students want to memorize terms (LC, turn 9). Carter shares an LC which demonstrates the explanation he offered: he recounts a recent experience with a student who thought he understood the material even though he couldn’t explain it (turn 10). He tried to convey to the student that struggling is evidence of not understanding (FC, turn 11), but the student would not budge on their stance (LC, turn 11).

Yin asks for clarification on the situation, not understanding what it is that students search for online (turn 13). Wallace explains what his students are doing (LC, turn 14) and synthesizes what Courtney and Carter said about the situation, that students use the terms they have read about without understanding them (FC, turn 15). The conversation ends with encouragement for Yin to try the unit in her course (turns 16–18); Wallace shares his lived concept that students are getting things out of the unit despite the minor challenges he has described (turn 19).

This conversation is grounded in the group’s collective understanding of the magnetism unit and the pedagogy built into NextGenPET. In another type of class, for example, a traditional introductory physics class, it would not necessarily be a problem if students search the internet for how magnetism works. However, in the NextGenPET course, this behavior (potentially) interrupts the guided inquiry structure of the curriculum. Courtney is able to share her lived experience with the exact problem Wallace describes and with Carter’s contribution connecting the problem to the formal concept of epistemology, we say there is a concept developed in this conversation: that some students do not have a sophisticated understanding of what it means to know something in science, and this may underlie their internet searching behavior. The TxOTL’s concept development component captures development that is grounded in context-specific teaching challenges (as in the above example), as well as challenges that are universal across teaching settings. An additional example of concept development, one that is independent of the NextGenPET context, is provided in the Supple-mentary Material, Table s1.

### Meeting segment categories

The second piece in detailing *what* a conversation is about entails describing the broad topic/function of the conversation. This information is captured by the meeting segment category codes. We identified eight different meeting segment categories that collectively describe the range of conversations we have observed in FOLC meetings. These categories and their definitions are shown in Table 6. Note, if we determine that a pedagogical concept was developed during a meeting segment, then that meeting segment is automatically labeled as “Developing a Pedagogical Concept”; none of the other meeting segment categories involve concept development.

**Table 6:** The eight meeting segment categories and their definitions. The ordering in the table reflects the priorities of the NextGenPET research team; categories are ordered from least (top row) to most (bottom row) valued

Meeting segment category	Definition
Social Chit-Chat	People talk about their family, themselves, life outside of work; this can include talk about work in the broad scope (e.g., sharing where they are employed) as long as the talk is not tied to FOLC activities or teaching work in detail
Meta	Discussing the operation of the FOLC (e.g., How to use the Slack Workspace; What the agenda of the meeting is)
Logistics	Discussing “how to do something” in one’s teaching work, but the issue is not pedagogically motivated (e.g., how to upload homework to a learning management system; equipment issues)
Status Update	Updating people on how one’s class is going (e.g., where you are in the curriculum, what units you plan to cover, how many students are in the class, how a lesson went); a report on your teaching “condition” with **no underlying reason** for it **presented**; can also entail report of one’s *experience* with a teaching strategy/an update on something you tried in the past
Generating detailed descriptions and explanations for pedagogical problem	When people are reporting in depth on a clearly articulated pedagogical issue, i.e., there is a description of what has happened and some statement or conjecture about **why** it is happening or why they care about the “what”
Generating solutions to a problem *without a why*	Describing in detail what one did in class to address a particular pedagogical issue. The issue itself may be implicit. These are conversations where people are reporting how they run some activity or deal with some issue in the classroom, “how to’s” that are pedagogically motivated (e.g., how they use student assistants; how they use a particular teaching strategy; how they run an activity). **No explanation** is proposed for the problem or solution. The “problem” can be the underlying pedagogical problem that drove the need for the solution, or it can refer to problems or rationale associated with implementing the solution.
Generating solutions to a problem *with a why*	Same as above, except an **explanation** is proposed for the problem or solution. A “why” is provided; either: “Why this is a problem/ why we care about it” or a conjecture as to “why this problem is occurring” or “Why I use the solution I do”
Developing a Pedagogical Concept	The group collectively addresses a pedagogical issue by making links between lived and formal concepts, developing a more general pedagogical concept that applies to the situation at hand and a number of other future teaching situations. (The developed concept, while often previously known to individual member(s) of the group and the broader education community, is new to the group’s *collective* knowledge.)

### Nature of hierarchy in the taxonomy

The TxOTL is meant to provide a structure for describing the range of OTLs observed in a FOLC meeting. However, the meeting segment categories are *not ordered in terms of increasing OTL*. There are two reasons for this.

First, in some ways, it does not make sense to compare learning opportunities given that they can be so different in nature. For example, during Social Chit-Chat segments, participants have the opportunity to learn about their fellow group members, and in Developing a Pedagogical Concept segments, participants have the opportunity to learn some formal concept about teaching and learning which is grounded in lived classroom experience. Is this “better” or “more” of an OTL than learning about your fellow members? These OTLs are very different in nature, one being non-pedagogical and one being pedagogical. Even when comparing two pedagogical OTLs, it is not clear how to assign value to the different OTLs. In Generating Solutions conversations, participants have the opportunity to learn about various solutions to a pedagogical issue. This is not necessarily “less” of an OTL than when the conversation extends to develop a pedagogical concept and rises to a Developing a Pedagogical Concept segment category. After all, if one is facing a problem in their classroom, it may be much more useful to get timely advice on how to solve the challenge, rather than to consider the broader pedagogical implications of the situation.

Second, even meeting segments in the same category can have different OTLs because of the differences in the communicative approach used in the segments. The general topic or characterization of the OTL corresponding to each meeting segment category is shown in Fig. 2 and holds regardless of the communicative approach used in the conversation. However, the “breadth” of the OTL can shift with the way ideas are presented. Consider a conversation in which someone asks about a lab equipment issue which is distracting students from the main point of the lab activity. This person asks for ideas about how to solve this problem. If there is only one idea proposed, the communicative approach for how ideas are presented is “mono.” If instead there is a diversity of ideas presented (e.g., “I do X,” “I do Y”), that is low interanimation of ideas. If participants in the conversation directly compare and contrast the different solutions proposed, that is high interanimation of ideas. Whichever way the conversation unfolds, we would say there was an opportunity to learn solutions to this pedagogical issue; however, broader opportunities for learning are supplied by the multiple perspectives present in dialogic conversation. The effect of communicative approach on the OTL for a given meeting segment category means we cannot say certain meeting segment categories offer “more” OTL than another segment category. For example, a Generating Detailed Description segment in which multiple explanations for a pedagogical challenge are compared (high interanimation) does not necessarily offer “less” of a learning opportunity than a Generating Solutions segment where only one idea is presented for solving a pedagogical issue (mono).

The TxOTL helps us describe how OTLs may differ from each other, but it does not assign value to different OTLs. That is, the taxonomy does not tell one about “better” OTLs or “more” or “less” OTLs. We hold that all OTLs described by the taxonomy are valuable. The taxonomy does allow one to see when an OTL is broader, i.e., providing multiple perspectives or ideas.

All this said, users can apply a hierarchy to the *meeting segment categories* based on the kinds of conversations they value. This value judgment is influenced by the needs and goals of the FOLC (or another professional development group whose conversations the taxonomy is applied to). The TxOTL user assesses alignment between meeting segment categories and their program’s goals and participants’ needs and assigns value accordingly. This means that for some faculty development groups, they may most highly value conversations that generate solutions to teaching challenges that members face and may not care as much about developing the pedagogical concept knowledge of members. This group would rank order the categories differently than what is shown in Table 6, which represents the values assigned by the NextGenPET FOLC research team. Doing this would not mean the OTLs provided by Developing a Pedagogical Concept segments are “less” or “worse” than the OTLs provided by Generating Solutions segments, but just that the group cares more about generating the latter OTL type.

In both Fig. 2 and Table 6, we have ordered the meeting segment categories in terms of proximity to developing a pedagogical concept and degree of mobilization for future pedagogical work (both increasing as one reads down the Table). In the NextGenPET FOLC, we ultimately want the members to reach the stage of developing pedagogical concepts in their conversations, connecting their lived teaching experiences to the deeper issues and factors at play. We most highly value concept development because it mobilizes participants in their teaching work for a whole class of situations associated with the concept being developed. In other words, concept development positions the group with the tools and concepts they can deploy in a *variety* of future teaching situations (in the NextGenPET course and beyond). For the NextGenPET FOLC then, we place greatest value on Developing a Pedagogical Concept segments.

When coding a conversation for the meeting segment category, we apply the highest level category that fits, “highest” according to the chosen hierarchy of meeting segment categories. A conversation often starts as one meeting segment category and then evolves into another category before the purpose of the discussion shifts enough to count as a new segment. For example, a participant may begin with a status update of their class, recounting the curricular units they plan to cover. Another participant may then raise the challenge of how to choose what units to cover in the class. The group then might discuss different solutions to this challenge, offering how they choose the topics they will cover and comparing options. By the end then, the conversation has evolved into a Generating Solutions segment and that would be the meeting segment category we would apply to the segment overall. This analytic choice allows us to flatten some of the information contained in a meeting segment, reducing analysis time while capturing essential distinctions between conversations. FOLC meetings contain abundant, complex social interaction, and in coding segments based on the highest level meeting segment category that fits, we reduce the scope of interactions we have to be attuned to in favor of focusing attention on the proximity to developing a pedagogical concept a conversation reached, as this is a goal for the NextGenPET FOLC participants.

### Combining the taxonomy elements

The communicative approach, concept development, and meeting segment category coding of a conversation combine to describe the type of OTL provided by that segment. As described in the preceding section, we can characterize the OTL in general terms based on the meeting segment category, but the mono-dialogic dimension to communicative approach provides more information on the breadth or expansiveness of the OTL.

We can also consider the connection between meeting segment categories and communicative approaches. Recalling that concept development requires the linking of lived and formal concepts, we see that concept development can only occur when “different ideas are explored and worked on by comparing, contrasting, developing” (Scott et al. 2006) [p.611]. When this happens, the segment’s communicative approach will be coded as high interanimation. There is not any direct linking of concepts (lived with formal, lived with another lived, formal with another formal) with a mono communicative approach (only one perspective is considered), nor with a low-interanimation communicative approach (multiple perspectives are simply introduced, but not connected). Thus, Developing a Pedagogical Concept segments—defined by the occurrence of concept development—must be paired with a high-interanimation communicative approach. High interanimation of ideas can be accomplished by one or multiple people, so the Developing a Pedagogical Concept segments can include one or multiple voices (non-interactive or interactive). The other seven meeting segment categories do not involve concept development, and as such they can (theoretically) appear with any combination of communicative approach codes. The theoretically possible combinations of taxonomy codes are depicted in Table 7.

**Table 7:** The theoretically possible combinations of TxOTL codes

Concept development	Communicative approach	Meeting segment category
No	N/A	Social Chit-Chat
	Mono, low interanimation, or	Meta
	high interanimation	Logistics
	interactive or non-interactive	Status Update
		Generating Detailed Description and Explanations for Pedagogical Problem
		Generating Solutions to problem without why
		Generating Solutions to problem with why
Yes	High interanimation	Developing a Pedagogical Concept
	interactive or non-interactive	

Concept development requires that a high interanimation communicative approach is used; however, high interanimation can occur when there is no concept development. For example, participants can compare and contrast lived concepts (such as their experience with a teaching issue), without discussing a formal concept. We do not code Social Chit-Chat segments for communicative approach (as explained in “Analytic approach” section)

We can also consider the combinations we have seen empirically, based on our coding of six FOLC meetings using the TxOTL. For example, in this data sample, we have seen Meta meeting segments paired with every communicative approach combination except non-interactive, high interimation. For Generating Solutions with a Why segments, we have seen every communicative approach except non-interactive, low interanimation. While we cannot generalize these results to other contexts, the fact that we have seen empirically many different combinations of meeting segment categories and communicative approaches confirms that both elements of the taxonomy are capturing important information. If we only coded for the meeting segment category, we would be missing an informative level of detail regarding the OTLs provided by conversations; communicative approach coding adds to our understanding of the OTL.

## Using the TxOTL

Having presented the entire TxOTL, we now turn to how it can be used. We start by describing the analytic approach for applying the tool to a FOLC (or other teacher workgroup) meeting. We then demonstrate how we compactly represent the taxonomy coding for an entire meeting. We provide examples of the taxonomy-in-use and the claims that the tool allows one to make.

### Analytic approach

Starting with a transcript of a workgroup meeting, we segment the meeting based on shifts in conversational purpose (Schegloff 2007). As Schegloff explains, “A great deal of talk-in-interaction - perhaps most of it – is better examined with respect to action than with respect to topicality, more for what it is doing than for what it is about.” When we determine the purpose of a segment, we are considering what the function of the segment is, i.e.,“what it is doing.”

Having segmented a meeting, we code each segment according to the following three-step process:

1. Code segment for communicative approach. If the communicative approach includes high interanimation, proceed to step 2. If not, proceed to step 3.
2. Identify and list the lived and formal concepts apparent in the high interanimation segments (while all segments can potentially have lived and formal concepts stated, concept *development* can only occur if the communicative approach of the segment involves high interanimation of these ideas). Next, consider how (and if) these concepts are connected. If they are clearly linked together, articulate the concept that is developed in the conversation. If a connection between the LCs and FCs cannot be identified, mark the segment as “no concept development.”
3. Select a meeting segment category that fits the segment. If in step 2 concept development was identified, the meeting segment category for the segment is Developing a Pedagogical Concept (note, one likely will have an idea of the segment category as they proceed through the first two steps, but they should not make a final assignment until they have done the communicative approach and concept development analysis.)

An exception to this process is if the meeting segment category is Social Chit-Chat. These segments are easy to identify (and at least for the NextGenPET FOLC, most frequently occur at the beginning and end of a meeting). As one reads over a segment, if they immediately identify it as Social Chit-Chat, they can proceed directly to step 3 and label it as such. We do not bother coding the communicative approach for this meeting segment category because it is at the bottom of the hierarchy for NextGenPET FOLC meetings, i.e., it has the farthest proximity from developing a pedagogical concept (however, if one places Social Chit-Chat higher in their hierarchy of meeting segment categories because their main goal, for example, is community formation, they may care more about the communicative approach used in this meeting segment category).

**Fig. 3:**
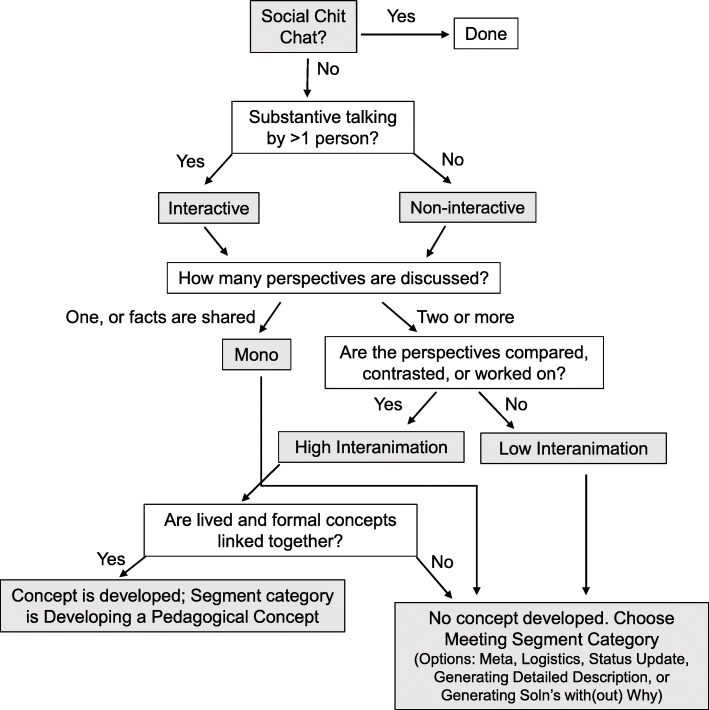
Flow chart representing the analytic process of applying the TxOTL to a meeting segment. Gray boxes include the taxonomy categories

The analytic approach for applying the taxonomy to a meeting segment is summarized in Fig. 3.

Once one has followed steps 1–3 for a given segment, they proceed to the next segment of the meeting and go through steps 1–3 again. One should work through the segments in sequential order. This is important because later parts of a meeting may refer to an earlier conversation in the meeting, and if one does not analyze the segments sequentially, they may miss essential context for understanding a segment. This point also raises the special case of segments that are discontinuous in time. Sometimes a conversation clearly refers to an earlier conversation in a meeting, and in these cases, if one determines the purpose of the two conversations to be the same, they count the two conversations as one segment and code them identically. Determining if the later conversation is a follow-up on the original conversation and if they have the same purpose is a judgment call based on the context of the conversation, the history of the group and previous meetings, and the dynamics between participants; one also looks for discursive markers to aid in this decision. For example, sometimes a participant will actually say, “Following up on the previous discussion about ___.” The process of applying the taxonomy to an entire 1-h meeting can range from approximately 1.5 to 4 h, depending on one’s experience coding with the TxOTL and the types of conversations contained in the meeting.

There are two general rules to apply when deciding how to code a meeting segment. First, as previously touched on, we always consider the context and framing of a conversation. The same thing said in different contexts could be coded differently (particularly when determining if something is a lived or formal concept). This emphasis on context is grounded in our sociocultural approach to learning. Second, we use what Horn et al. call the “engaged newcomer” heuristic (Horn et al. 2017) (described in “Concept development” section.). This prevents us from over-interpreting a conversation and drawing conclusions that a new member would be unlikely to make in-the-moment of the conversation.

### Timeline representation

**Fig. 4:**
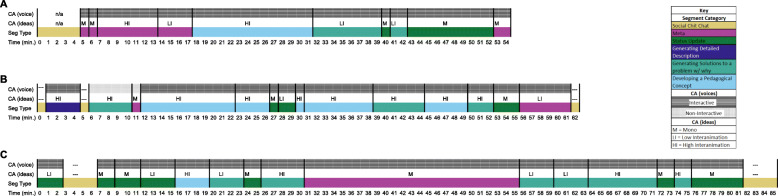
**A** A timeline representation of a FOLC meeting coded with the TxOTL. The vertical black lines divide the meeting into segments. The bottom line tracks the time in the meeting. Reading the timeline from left to right shows how the codes were assigned to each segment during the meeting. Reading the timeline vertically shows how a particular segment was coded along all the taxonomy dimensions. **B** Timeline representation of another NextGenPET FOLC meeting coded with the taxonomy. **C** Timeline of a second meeting from the same NextGenPET FOLC group represented in timeline B

Once we have applied the TxOTL to each segment in a meeting, we construct a “timeline image” of the meeting in order to draw inferences and interpret the coding results. This timeline representation shows in a condensed form how each segment in a meeting was coded along the different taxonomy constructs. One example is shown in Fig. 4-timeline A. The bottom line of the figure tracks the time in the meeting. Segments are demarcated in the figure by vertical, black lines. The meeting segment categories are color-coded, as shown in the line above the time markings. The color palette used is from (Nichols) and is designed to be color-deficiency accessible. In the line above the meeting segment categories, we mark the mono-dialogic dimension coding; this row is labeled as “CA (ideas)” because the mono-dialogic dimension of communicative approach (CA) describes how ideas are discussed. We use initials to mark the codes under this dimension: “M” standing for mono, “LI” standing for low interanimation, and “HI” standing for high interanimation. The top line of the figure represents the interactive-non-interactive dimension coding; this row is labeled as “CA (voice).” The light, dot-pattern represents non-interactive segments, while the dark, horizontal line pattern designates interactive segments. Reading the timeline from left to right shows how the codes were assigned to each segment during the meeting. Reading the timeline vertically shows how a particular segment was coded along the three taxonomy dimensions.

### Interpreting a timeline representation

We will now explore the types of inferences and information we can draw from a timeline representation of a meeting coded with the TxOTL. Figure 4-timeline B shows the timeline for a meeting from one of the NextGenPET FOLC groups. What claims can we make about this meeting? Looking at the top row of the timeline, it appears that there were a number of voices heard in this meeting; all but two segments are coded as interactive (ignoring the Social Chit-Chat segments where we do not attend to communicative approach). Next, reviewing the communicative approach (ideas) row, we can see that the majority of the meeting segments included the presentation of multiple perspectives (LI or HI), and furthermore, most of the segments involved participants comparing and contrasting the perspectives introduced (HI segments). Finally, the meeting segment category row tells us that the meeting started and ended with Social Chit-Chat, and in the middle most of the conversational segments involved Status Updates, Generating Solutions, or Developing a Pedagogical Concept. Note, however, “most of the conversational segments” is not the same as “most of the time.” Because we code a segment based on the highest-level code that fits, we cannot make claims about the time spent in different modes. It would be incorrect to say that half of the meeting time shown in Fig. 4B was spent on Developing a Pedagogical Concept; while a concept was developed in each of the five Developing a Pedagogical Concept segments, the conversations likely started as a Status Update or Generating Solutions and evolved to develop a concept. That is, in a 10-min segment coded as Developing a Pedagogical Concept, it may be that the first 8 min were spent on Generating Solutions to some problem and only in the last 2 min were formal and lived concepts clearly linked together, resulting in concept development and raising the conversational segment to Developing a Pedagogical Concept. Thus, we are attending to frequencies (counts) of codes when making statements about “least” or “most,” rather than time.

The timeline also indicates areas to explore further. For example, the only non-interactive portion of the meeting happened in minutes 6–11. We may want to revisit those meeting segments to see who was talking and what the facilitators were doing during those segments. It is also interesting that from minutes 6–10, the one person talking introduced multiple ideas on a topic and compared them (HI). This indicates that the person who was speaking may have a lot of resources to draw on, and they may have a big influence on the direction of conversations. Thus, the timeline representation has analytic value to researchers wanting to explore the group dynamics and learning opportunities in FOLC meetings, but also practical use to the meeting facilitators who want to learn how to encourage participation and various types of discussions.

We can also explore meeting trends and conversational patterns of a FOLC group by comparing timeline representations. The timeline in Fig. 4B represents just one meeting of a NextGenPET FOLC group. The TxOTL can be applied to many more of this group’s meetings and a corresponding timeline representation can be produced for each meeting. To illustrate the types of comparisons, the taxonomy (and corresponding timeline representation) can facilitate, consider Fig. 4-timeline C; timeline C is a timeline of the meeting following the one depicted in timeline B, from the same NextGenPET FOLC group. From timeline C, we can see that the group again had a meeting where the majority of the segments (actually all of them) were interactive. While the majority of meeting segments in Fig. 4B are labeled with a high-interanimation communicative approach (ideas), the ways ideas were discussed in the meeting depicted in Fig. 4C were more evenly distributed between the three modes (M, LI, HI). We also see that the meeting in Fig. 4C is dominated (in terms of frequency) by Status Update and Generating Solutions to a Problem with a Why conversations and has only one Developing a Pedagogical Concept segment. In contrast, the meeting in Fig. 4B had five Developing a Pedagogical Concept segments. These differences in meeting segment categories and the ways ideas were discussed (CA ideas) are consequential for the types of learning opportunities provided by the meetings. It seems the meeting represented in Fig. 4C was more focused on practical applications to specific problems than higher-level principles or generalizations about teaching and learning. We want our FOLCs to discuss practical issues, so it is good we see this, but we would also want to investigate the framing of this meeting by the facilitators to see if that was consequential for the meeting segment categories that ensued, and also if there were facilitation moves used in the meeting in Fig. 4B that pushed more of the conversations to the level of Developing a Pedagogical Concept.

Of course, we cannot make general claims about this NextGenPET FOLC group after comparing just two meetings, but if the taxonomy was applied to a larger sample of their meetings, more robust claims about their communication patterns and the OTLs provided in this group’s meetings could be developed. For example, one could explore what types of communicative approaches are used by the group during a given meeting segment category. One could also examine if the group has a dominant communicative approach across meeting segment categories. It may further be useful in tracking shifts in meeting segment categories’ frequencies over time. This would inform one about how the nature of the OTLs provided by the FOLC meetings evolve for the group. For a group’s facilitator, this information would also allow them to see if their group’s meetings tend to be dominated by meeting segment categories that align with their main goals for the group, and if this is not the case, they can course-correct. The TxOTL’s value for facilitators is further discussed in “Facilitator perspectives on how the TxOTL can inform their professional development practice” section.

### Value and limitations of timeline representation

The timeline representation provides a compact visual summary of the results from applying the TxOTL to a FOLC meeting, but in order to generate this representation, someone has to code the meeting with the taxonomy. Through this coding work, one gains a detailed and direct sense of the conversations in the meeting and the OTLs provided. What, then, is the added value of the timeline representation?

First, the timeline representation provides an overview of what occurred in a meeting, containing more detail than one can keep in their head at once when analyzing a transcript from an hour-long meeting. This overview presentation makes it easy to quantify the elements captured by the taxonomy (e.g., how many segments are coded with a given communicative approach). Second, given the compactness of these representations, they also allow one to “see” multiple meetings at once, facilitating the comparison of meetings from a given group over time. Additionally, the timeline representation simultaneously provides a bird’s eye view of a meeting while helping to identify areas of a meeting to explore in further detail. For example, one may be interested in the *variety* of solutions group members offer for a given pedagogical problem, and through the timeline representation, they could quickly identify the portions of a meeting transcript they will need to read to answer this question (namely, Developing a Pedagogical Concept segments or Generating Solutions segments with a communicative approach involving low or high interanimation). The timeline representations are a tool for asking questions about a meeting.

All of the above advantages of the timeline representations are afforded to both the person who produces the representation, as well as to a person who is handed a timeline someone else produced. For this latter group, the timeline representation can help them explore their own questions, given that they understand the taxonomy codes and the assumptions built into the tool. The questions from this group may be distinct from those of the person who produced the representation. The timeline representations help transfer or distribute the analysis of a meeting from one group of people to another, and from one set of research questions to another.

With all this said, it is also important to acknowledge that the timeline representation, in providing a compact overview of a meeting coded with the TxOTL, omits a level of detail that is retained in the original transcript or recording. The data reduction represented by the timelines affects the level of comparison one can make between FOLC meetings. For example, one can compare the frequency of concept development segments that occurs in two different meetings based on their timeline representations, but if one wanted to compare the content of the concepts developed, they would have to return to the transcripts of the meetings. Similarly, if one was interested in comparing the time spent connecting lived and formal pedagogical concepts during different meetings, one would have to return to the transcript to count the exact time because of our decision, reflected in the timeline representation, to code based on the highest-level meeting segment category that fits. The timeline tells us about the *frequency* of, *not time* spent in, different meeting segment categories.

### Facilitator perspectives on how the TxOTL can inform their professional development practice

As mentioned in “Member-checking interviews” section, in order to include the voices of the FOLC facilitators in our research process, we conducted interviews with a sample of four of the current ten NextGenPET FOLC facilitators. These interviews provide insights about how the TxOTL can help facilitators of professional development programs develop their practice. Overall, all four interviewees thought the timeline representation of meetings coded with the taxonomy provided useful information. One facilitator talked about how the first thing he would look for in the timeline was the line that tells us about communicative approach-voices: if segments were interactive or non-interactive. As a facilitator, he wants to be attuned to how many voices are heard. This facilitator also said they would be interested in seeing trends between groups, within the same group over time, and between codes. As we have shown above, the taxonomy affords these types of comparisons. Another facilitator expressed that the timelines could serve as a tool to provide facilitators with a suite of “good” meeting examples, where the meetings could be “good” along different dimensions of the taxonomy. Building on this facilitator’s idea, we could imagine using timelines of “good” meetings to train facilitators about what to focus on if, e.g., they want to increase the opportunities in their meetings to generate solutions to pedagogical problems.

Echoing the ideas of these two facilitators, another facilitator we interviewed recognized that the taxonomy could help one identify features which accompany the different types of conversations, and that this could be a great professional development activity. He suggested that facilitators and participants could look at meetings coded with the taxonomy and identify connections together. He thought this would be useful training for the facilitators of the meetings, but also for the participants because they often do not know what causes a conversation to be productive. (Note, though, to develop robust *explanations* for the patterns they identify they would have to return to the meeting transcript.) This feedback speaks to the practical utility of the taxonomy for those who are actually participating and leading these faculty groups.

In interpreting the timelines, one facilitator wondered about the transitions between meeting segment categories and what drives that. For example, he noticed that in one of the timelines Generating Solutions segments seemed to precede Developing a Pedagogical Concept segments, but in the other timeline, we provided there was the opposite pattern. The timeline pinpoints areas of a meeting to explore further and would allow one to examine this facilitator’s question. This could then inform a facilitator’s actions in a meeting as they seek to guide conversations toward specified ends.

Finally, the facilitator who suggested using the taxonomy for a professional development activity also said that this tool may be able to identify conditions that lead to concept development in conversations. He said this could be very powerful information for professional development programs because in his experience, it is often hard to get to a concept development stage in professional development workshops. Indeed, if one cares about opportunities for participants to reflect deeply on lived and formal concepts, then it is essential for the facilitator of the professional development to know when in a meeting these opportunities are occurring and what meeting patterns surround these conversations; the TxOTL tells you this information. This facilitator’s point also speaks to the broader impact of the TxOTL; it has the potential to inform the practices of different forms of professional development. It is interesting to consider what “lessons learned” from applying the taxonomy to FOLC meetings (e.g., conditions that lead to certain meeting segment categories) would potentially apply to other professional development environments. (This will be discussed further in “Applicability and limitations” section.)

## Discussion

In this section, we consider the broader applicability of the TxOTL. The TxOTL was developed and tested in two FOLC environments, the NextGenPET FOLC and NFW-FOLC. In considering the applicability of this tool to other contexts, it is important to first acknowledge what aspects of the professional development environment the taxonomy is attuned to and those aspects that it backgrounds.

### Focus of the TxOTL

The taxonomy provides a structure for describing the learning opportunities in different workgroup conversations, capturing information on the nature and content of the discourse. This tool focuses attention on only four of the myriad social dynamics contained in a 1-h meeting: the way ideas are presented, the interactiveness of conversation, the concepts participants draw on and develop, and the broad-scale function of a conversation. The TxOTL allows one to explore patterns and connections between these elements of a group’s conversation. We focus on these four elements because they are consequential for the OTLs provided in a conversation.

With a focus on OTLs, the taxonomy foregrounds concept development that occurs in conversation. Recall that OTLs are operationalized by considering how ideas are discussed, how a conversation mobilizes people for future work, and what conceptual resources are used in the conversation (Horn and Kane 2015; Horn et al. 2017; Horn et al. 2015). In applying the TxOTL to a FOLC meeting, one is guided to attend to the conceptual resources (particularly, lived and formal concepts) being used. Even if the user imposes a different hierarchy of meeting segment categories than the one we have adopted, concept development (and identifying lived and formal concepts) is still an aspect of conversation that the taxonomy guides one to examine. We have built into the TxOTL this focus on concept development because in the process of a group developing a pedagogical concept, they must reflect on their practice and this is an important goal of STEM faculty professional development (Manduca 2017), including that of the NextGenPET FOLC. Additionally, by its very nature concept development mobilizes a faculty member to handle a number of future teaching situations.

With the foregrounding of concept development, the TxOTL backgrounds other outcomes one may be interested in. For example, this tool is not designed to capture the self-efficacy of a participant in problem solving around a teaching dilemma or the agency with which participants are positioned and afforded in a conversation. The TxOTL can help study these elements of workgroup interaction, but it is likely not the most direct or relevant tool to apply. Taking the example of the agency of participants in a conversation, communicative approach coding captures how ideas are considered and if more than one person is talking, but because the taxonomy is attuned to the OTL available to the group as a whole, details about *individuals* are not automatically captured. Similarly, if one is mainly interested in community formation in a teacher workgroup, they can place Social Chit-Chat and Meta segments at the top of their hierarchy of meeting segment categories, but more detail on individual interactions will be needed than is captured by communicative approach coding, and, in that case, concept development coding would likely become extraneous information.

The focus on *opportunities to learn* also means that the taxonomy is not meant to capture what a faculty member goes and does outside of their workgroup meeting. Instead, the taxonomy focuses attention on the learning environment of the professional development setting (e.g., a FOLC meeting) where faculty are engaging in the *process* of learning. It is in this setting that faculty are exposed to new pedagogical ideas that they can later act on. Opportunities to learn represent one key step in the process of faculty changing and developing their pedagogical knowledge and behavior. The taxonomy tells us about these OTLs but does not comment on the outcomes of these opportunities.

### Applicability and limitations

With this knowledge of the elements the TxOTL foregrounds and backgrounds, we can speak to its broader applicability. As described in “Tool refinement” section, we have one test of the external validity of this tool (beyond the NextGenPET FOLC) from applying it to the NFW-FOLC. Through our training of a new research team member and our interviews with facilitators, we also have proof-of-principle that members outside the original research team can gain shared understanding of the taxonomy elements, consistent with that of the original team. Based on these experiences, we expect the taxonomy to be applicable to other STEM teaching professional development contexts if (1)the program has goals similar to those of the NextGenPET FOLC and NFW-FOLC and (2)the questions one asks are aligned with the information the taxonomy is positioned to capture.

Regarding the goals of the professional development program, the NextGenPET FOLC and NFW-FOLC both focus on helping faculty members implement research-based instructional strategies through providing troubleshooting as issues are encountered in the classroom and supporting instructors’ development of reflective skills. The TxOTL should apply to other FOLCs that have similar goals, even if they are focused on a different group of faculty or different curriculum/pedagogical technique. Additionally, the taxonomy should be useful for studying in-person faculty learning communities (FLCs). Afterall, FOLCs are based on the (in-person) FLC model and as such they derive their goals from those of a FLC; FLCs and FOLCs share a focus on building a long-term professional development community which provides OTLs around teaching and develops the reflective practice of participants. None of the taxonomy elements depend on the modality (i.e., in-person vs. virtual) of the professional development environment under study.

In contrast, it is not readily apparent that the TxOTL would be applicable to single or multi-day workshops. One-time workshops can introduce faculty to a range of teaching techniques or provide intensive training in a specific technique, but given their format they do not have the goal of providing teaching support over the long-term, contemporaneous with instructors’ time in the classroom. As a one-time intervention, they also have limited capacity to help teachers develop reflective skills, and they are much less focused on community formation. Given these differences in purpose, many of the meeting segment categories in the taxonomy would be inapplicable to a workshop setting. For example, in a one-time workshop we would not expect to see many Meta conversations, navigating how the community functions, because the community formation is limited. The overall framework of considering the content of a conversation and how participants engage in the conversation is useful for studying professional development workshops, but there exists a tool pre-dating the taxonomy (the Real-Time Professional Development Observation Tool (R-PDOT) (Olmstead and Turpen 2016)) that utilizes this structure and was specifically developed to study workshops. The categories in the R-PDOT that capture the content of a workshop (i.e., the topics and activities) are likely more relevant and useful for studying a workshop environment than the meeting segment categories defined in the TxOTL.

The taxonomy is unlikely to apply to a K-12 teacher professional development setting. Recall, we had to greatly adapt and modify Horn et al.’s taxonomy that they developed in the context of middle school mathematics teacher workgroups (Horn et al. 2017) in order to fit the higher-education context of the FOLCs. One of the main changes we had to make was to the meeting segment categories because the content of the middle school math teacher workgroup meetings and that of the NextGenPET FOLC and NFW-FOLC meetings greatly differed given the different constraints and concerns of the instructors. Thus, we would not expect many of our meeting segment categories to apply to K-12 teacher workgroup meetings. That said, the communicative approach codes we use (adapted from (Mortimer and Scott 2003)) capture more detail than the categories in Horn et al.’s tool, and they could translate to the K-12 teacher workgroup context.

In considering applicability, one also has to consider the interests of the researcher or professional development practitioner. The professional development context may have goals aligned with that of the NextGenPET FOLC, but if the person studying that environment does not care about group-level OTLs or concept development, the taxonomy is likely not the best tool for them to use. As discussed above, the taxonomy does not explicitly capture information about individuals and outcomes such as agency around problem solving about teaching. Thus, the taxonomy is not well-suited to answer questions about these topics. Additionally, the taxonomy was developed to be a reflective tool to be applied after a meeting occurs, rather than in real-time; if a user wants a tool to study their professional development context in real-time, the R-PDOT or another instrument would be better than the taxonomy. Lastly, the taxonomy helps one analyze *within* a given professional development context, rather than *across* contexts. This means that for someone hoping to compare professional development environments, the taxonomy can be a good tool to use if the two contexts are very similar in structure and goals (e.g., two NextGenPET FOLC groups) and if they share the same hierarchical valuing of meeting segment categories. If this is not the case, the taxonomy is of limited use for the purpose of comparing the environments.

## Conclusions

In this paper, we have presented a taxonomy for characterizing opportunities to learn in faculty (online) learning community meetings, the TxOTL. We have established what types of questions the taxonomy is well-positioned to answer, and those it is not so attuned to. It allows one to talk about group-level OTLs, analyze concept development, track change over time in a given context, and identify patterns between meeting segment categories and communicative approaches. It does not capture some of the fine-grained detail on conversational dynamics (e.g., names of speakers), speak to an individual’s learning, or focus on outcomes such as self-efficacy. The taxonomy is most applicable to FOLCs and in-person FLCs, with limited use for one-time workshops and K-12 professional development contexts.

The TxOTL is a powerful analytic resource for researchers to explore the social dynamics and affordances for OTLs that are provided by the increasingly prevalent professional development spaces to support STEM faculty’s teaching practice in higher-education. The TxOTL helps both researchers and facilitators identify patterns and aspects of meetings to examine further. The information gained from applying the taxonomy and exploring the questions it helps raise also has practical utility for the facilitators of these programs with the potential to guide their training. It informs them about the OTLs present in their professional development meetings and what OTLs are missing. A major focus of our future work with the taxonomy will be to examine its use and efficacy as a training tool for professional development facilitators.

## Supplementary Information

**Additional file 1** Supplementary Material.

## Data Availability

Meeting recordings and transcripts are not publicly available due to confidentiality concerns.

## Abbreviations

FLC: Faculty learning community
FOLC: Faculty online learning community
NextGenPET: Next Generation Physical Science and Everyday Thinking
NFW: New faculty workshop
RBIS: Research-based instructional strategy
OTL: Opportunity to learn
TxOTL: Taxonomy of Opportunities to Learn

## Footnotes

1 Both lecture and studio versions were designed using similar principles for student learning, but are implemented differently due to the differences in classroom setting, meeting time, and available resources. A studio classroom is designed for students to work on activities and experiments in small groups.
