# Supplementary material for: The Taxonomy of Opportunities to Learn (TxOTL): a tool for understanding the learning potential and substance of interactions in faculty (online) learning community meetings
Source: Int J STEM Educ. 2021 Jul 17;8(1):45. doi: 10.1186/s40594-021-00301-3 (PMC8285718; doi:10.1186/s40594-021-00301-3)
Supplement: Supplementary file 1 — Additional file 1 Supplementary Material. [file 40594_2021_301_MOESM1_ESM.pdf]

Supplementary Material

In the main text, we provided an example of concept development in a conversation that depended on the specific context (the shared curriculum) of the NextGenPET FOLC. Here we provide an additional example of concept development, this time with a NextGenPET FOLC conversation that did not directly depend on their shared curriculum. Concept development can occur around context-specific teaching issues as well as more universal pedagogical issues.

In Table s1 we include the transcript of a five minute excerpt from a ten minute conversation in one of the NextGenPET FOLC groups. The conversation was opened by Courtney, one of the group's leaders, asking the group, "So how do you guys give tests? How many tests do you give? When do you give your first test?" For the next five minutes, group members share their lived concepts (i.e. lived experiences): the number of exams they give, if they give a final, when their first exam comes about, and how far in advance they schedule exams. Then the conversation reaches what is shown in Table s1. Yin, agreeing with what others have shared, says that at the start of the term she only gives an estimate of when the exams will be (her lived concept, LC, Turn 1 in Table s1). She then asks for people's thoughts on giving two midterms during her course (she is on the quarter-system). Courtney is interested in this question and prompts Yin to share more about what she has done in the course. Yin says that the first time she taught the course she gave frequent quizzes and she didn't do this the second time she taught the course (LC, Turn 3). She then states a formal concept (FC, Turn 3) that more frequent quizzing keeps students more engaged with the material. She also posits that a difference in the class population could account for the difference in engagement; we don't label this as a FC though because the underlying concept (that no two classes are identical and there is some natural variation in how they respond to the course) is too implicit for an engaged newcomer to learn from.

Table s1: Transcript of the last five minutes of a ten minute NextGenPET FOLC conversation about assessment practices. All names are pseudonyms. Formal concepts are marked in Red and lived concepts in Blue. Turns of talk represent a continuous flow of conversation.

Turn	Speaker	Transcript
1	Yin	No, I was wondering, don't have a schedule. I totally understand that. I only gave an estimate date of exam, but would it also be two midterms? Does that sound typical or reasonable?
2	Courtney	I mean, I think so. Actually, that's what I was interested in. What do you do?
3	Yin	I do notice what makes a difference is the first time I taught it I gave frequent quizzes. Really short quiz, a five minute quiz throughout, four quizzes throughout the quarter. The second time I teach it, I taught it and did not give quiz, only the midterm. I felt maybe having the quiz is keeping students a little bit more engaged, or could it be I just happened to have a different group of population the second time. I don't know. Maybe the third time I'll know the answer.
4	Carter	Yeah. I give just the one summative test at the end of each unit. And I do have the sense that the students have a false sense of their own understanding based on the homework and things. So when they get to the quiz, there are some students who don't perform as well as they think they were going to, or they feel somehow surprised. So I'm wondering if giving earlier, shorter, low stakes quizzes, but where they can really see when they're by themselves without being able to talk to their neighbor, or look in their book, or whatever, here's what you know and are able to do. The first time they find that out is four weeks in, when they're taking the exam at the end of the energy unit.
5	Wallace	So in my introductory physics class, last semester, I moved to giving five midterm tests and a final and the first test at the end of the second week for exactly this reason. Last year, I've been trying to hammer on at students about metacognition and thinking about their own learning because, as you said, if you ask them before a test how they think they're going to do and compare with how they actually do, it's very few of them have a good idea of their own understanding. And I've only done it one semester, but it seemed to work pretty well. I think they were lower stakes, shorter tests, but they were able to ... A lot of them did pretty badly on the first test, but that in itself made them take more seriously what I was trying to tell them about the various adjust your study habits, and think about how you do things like read the textbook, and so on.

Turn	Speaker	Transcript
6	Wallace	So I was thinking about testing more frequently as well in this class. And I think this is, since I'm teaching future teachers, they're obviously a good population ... They should be more receptive to things like metacognition, and [crosstalk] this should tie into what they learn in their education classes. So that means I'm going to be testing them probably twice per unit.
7	Carter	Okay.
8	Wallace	So I have yet to figure out the details of that, but that's what I'm thinking of.
9	Carter	In these courses, and actually PET courses where I think for most of us they're studio style, right? And there are longer periods of class meeting. It seems fairly reasonable to have a class meeting where part of the class is spent on doing curriculum, but then a part of it is spent on testing. So that if you decide to give more tests during the term, you don't have to waste more classes. Whereas with their typical 50 minute introductory course, if you want to give five midterms, I would imagine it'd be hard to recoup, even if the tests are shorter than they otherwise would be, it might be difficult to recoup the time on that particular day to do any more lessons. I don't know, but that's appealing.
10	Courtney	Yeah.

Carter then adds in his LC, that he only gives end-of-unit tests (Turn 4). He makes a generalization about students in the course, noting that they do not have a good sense of their own learning (FC, Turn 4). He supports this claim with an LC that students are often disappointed in how they perform on the exams, thinking they will do better than how they actually perform. He offers the conjecture that "earlier, shorter, low stakes quizzes" will help students better gauge their own understanding (FC, Turn 4).

Wallace builds on what Carter has shared, saying he instituted frequent tests the last time he taught his introductory physics course (LC, Turn 5). He explains this choice with the FC of metacognition (Turn 5); like Carter, he discovered that students have a hard time judging their own learning, based on their poor predictions of how they will perform on a test (LC, Turn 5). He says that the frequent, lower-stakes tests seemed to help his students adjust their study habits after the first test where they did not perform well (LC, Turn 5). He then rehearses his plan for his NextGenPET course, saying he will try the frequent testing in that course as well (LC, Turn 6). Re-emphasizing the formal concept of metacognition (Turn

6), he says this should resonate with the NextGenPET students because they are future teachers and should be familiar with the concept. Wallace thinks he will be testing two times per unit in the NextGenPET course, but he is still working out details (LC, Turn 6 & 8). Carter ends the conversation, saying that in a studio-style NextGenPET course, there is enough class time to have frequent tests (LC, Turn 9). The group then shifts to a new topic of discussion.

We say there is concept development in this conversation because Yin, Carter, and Wallace directly connect their classroom experiences around the frequency of testing to the formal concepts of students' metacognition and engagement. From this conversation, the group has the opportunity to learn the pedagogical concept that regular, low-stakes testing helps students gauge their understanding, develop metacognitive skills, and stay engaged.
